# Microwave-assisted Facile and Ultrafast Growth of ZnO Nanostructures and Proposition of Alternative Microwave-assisted Methods to Address Growth Stoppage

**DOI:** 10.1038/srep24870

**Published:** 2016-04-22

**Authors:** Abu ul Hassan Sarwar Rana, Mingi Kang, Hyun-Seok Kim

**Affiliations:** 1 Division of Electronics and Electrical Engineering, Dongguk University-Seoul, Seoul 04620, South Korea

## Abstract

The time constraint in the growth of ZnO nanostructures when using a hydrothermal method is of paramount importance in contemporary research, where a long fabrication time rots the very essence of the research on ZnO nanostructures. In this study, we present the facile and ultrafast growth of ZnO nanostructures in a domestic microwave oven within a pressurized environment in just a few minutes. This method is preferred for the conventional solution-based method because of the ultrafast supersaturation of zinc salts and the fabrication of high-quality nanostructures. The study of the effect of seed layer density, growth time, and the solution’s molar concentration on the morphology, alignment, density, and aspect ratio of ZnO nanorods (ZNRs) is explored. It is found in a microwave-assisted direct growth method that ~5 mins is the optimum time beyond which homogeneous nucleation supersedes heterogeneous nucleation, which results in the growth stoppage of ZNRs. To deal with this issue, we propound different methods such as microwave-assisted solution-replacement, preheating, and PEI-based growth methods, where growth stoppage is addressed and ZNRs with a high aspect ratio can be grown. Furthermore, high-quality ZnO nanoflowers and ZnO nanowalls are fabricated via ammonium hydroxide treatment in a very short time.

In recent years, ZnO has attracted special attention in research and development because of its unique optical, chemical, electrical, and piezoelectrical properties^1^,^2^,^3^,^4^. It has a myriad of potential applications in the realm of gas sensors, light-emitting diodes, field effect transistors, ultraviolet lasers, photodetectors, piezo-nanogenerators, and solar cells^5^,^6^,^7^,^8^,^9^,^10^,^11^. The eminent morphologies achieved from crystalline nanostructured ZnO are nanoparticles, nanotubes, tetrapods, nanorods, nanoflowers, and hemispheres^12^,^13^,^14^,^15^,^16^,^17^. Some of the more prominent methods used to synthesize ZnO nanostructures are vapor-liquid-solid (VLS), metal-organic chemical vapor deposition (MOCVD), pulsed laser deposition, and thermal evaporation^18^,^19^,^20^,^21^. However, the use of very high temperatures (500–900 °C), a specially designed catalyst, and a limited choice of substrates are a few of the limitations of these methods. However, solution growth methods are more energy efficient and cost effective, and can be used in a large scale for the growth of ZnO nanostructures^22^,^23^,^24^,^25^. Hydrothermal is the most prominent solution growth method, where zinc nitrate hexahydrate [Zn(NO_3_)_2_·6H_2_O] and methenamine [C_6_H_12_N_4_] are used as a precursor solution at temperatures as low as 80–100 °C.

The conventional convective hydrothermal methods of fabrication are too lengthy and time-consuming^26^. Time is invaluable and its importance is furthered in such fabrication processes where one has to progress many trial-and-error experiments to obtain optimum results. In this backdrop, microwave synthesis is a landmark in rapid volumetric heating with small reaction time and high reaction rate, selectivity, and yield. Conventional heating techniques take much longer to reach the crystallization temperature via convection, hence leading to a thermal gradient throughout the bulk media and to inefficient and non-uniform reactions, which may create serious issues for the crystal growth^27^. The most important mechanisms involved in microwave heating are dipolar polarization and ionic conduction by dint of dielectric heating. In the proposed microwave-assisted hydrothermal method, water is used as a dipolar solvent and heat is produced by the rotation, friction, and collision of water molecules under the influence of a rapidly changing alternating electric field. Furthermore, the dissolved ions in the solution move constantly under the influence of the fluctuating electric field, causing a steep ascent in the local temperature due to friction and collision.

In this study, we use an 850 watts (2.45 GHz) domestic microwave oven for the growth of ZnO nanostructures. The ultrafast crystallization of ZnO nanostructures can be achieved in only a few minutes, which take hours otherwise. The ultrafast supersaturation and homogeneous nucleation of zinc salts under microwave irradiation are checked and alternative methods such as microwave-assisted replacing solution, preheating, and PEI-based growth methods are also propounded as potential solutions to growth stoppage. Furthermore, high-quality ZnO nanoflowers (ZNFs) and ZnO nanowalls (ZNWs) are fabricated in amazingly short fabrication time via ammonium hydroxide treatment. Every method proposed in this study has its own limitations, benefits, and research and development applications. SEM, XRD, EDS, and PL are exploited to examine the morphology, crystallinity, elemental setup, and luminescent properties of the nanostructures, respectively.

## Results and Discussion

### Microwave chemistry

Rapid heating under microwave irradiation was achieved via the dipolar polarization of water molecules and the ionic conduction of the dissolved chemicals. Microwave heating is preferred to conventional heating methods because of the homogeneous heat transfer to the solution mixture for chemical reactions. Irradiation raises the temperature of the whole solution simultaneously, which accelerates both nucleation and crystal growth^28^. Homogeneous heat transfer is further supported by the anticlockwise rotation of the disc inside the oven at 4 rpm. The chemistry of the microwave method is based on efficient heating rather than inducing chemical reactions via the direct absorption of electromagnetic waves, because the energy of 850 watts and 2.45 GHz microwaves, which is 1.0 × 10^−5^ eV, is not sufficient to cleave typical chemical bonds^29^.

**Figure 1:**
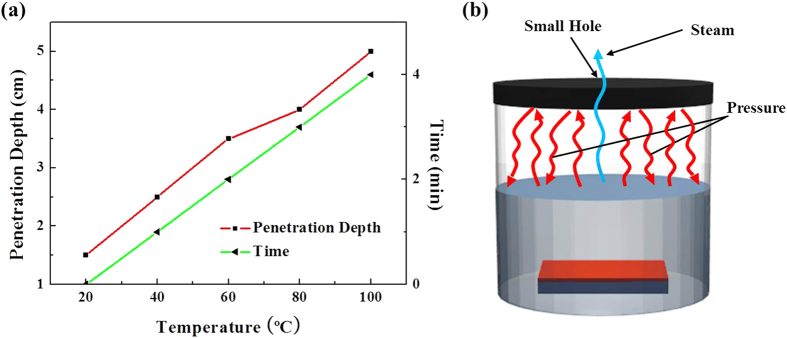
(**a**) Penetration depth of microwaves and the time vs. temperature of the solution upon reaching supersaturation, and (**b**) Specially designed reaction vessel for microwave-assisted growth methods.

The microwave heating also depends upon the volume and the depth of the solution. The penetration depth is the point at which 37% of the initially irradiated microwave power exists effectively in the solution^30^. The penetration depth of 2.45 GHz microwaves in water is 5 cm at 100 °C. If the volume and depth of the solution is above 5 cm, the remaining solution will be heated via convection rather than irradiation, which could alter the results. Hence, we chose a wide faced reaction flask of 300 ml volume where the height of the solution was markedly below 5 cm and the wide area of the solution was homogeneously exposed to the microwave radiation. Figure 1(a) shows the temperature of the solution versus the penetration depth and time, which is drawn as per the data taken via *in situ* temperature testing using a wired thermometer attached at the base of the solution. As the depth of the beaker was below 5 cm, the thermometer recorded the same temperature for the solution all over the beaker.

Besides rapid heating, another important factor in microwave-assisted hydrothermal methods is the existence of a pressurized environment inside the reaction vessel. To shy away from the rapid evaporation of the solution under high power microwaves, we use a closed reaction system rather than an open reaction system. The closed reaction system facilitates pressurized conditions that could raise the temperature of a solution well above its boiling point. Hence, the ultrafast chemical reactions are not only because of the elevated temperature, but also due to the pressurized conditions inside the vessel. The whole process is very sensitive, because any extra pressure created inside the sealed vessel could burst it with devastating results. For safety, the autogenous pressure inside the vessel could also be measured^31^, but to deal with the predicament, we used pulsed microwave heating and a specially designed reaction vessel with a small hole at the top, as shown in Fig. 1(b). Pulsed microwave heating helps control the temperature of the solution in the required range, and the hole not only emancipates the vessel explosion by wasting the extra pressure created inside the vessel in the form of steam, but also maintains the pressure required for the rapid flow of the chemical reaction and ultimately the ultrafast growth of ZnO nanostructures.

### Seed layer

The lattice mismatch between ZnO (a = 3.249 Å) and P–Si (a = 5.43 Å) is 2.19 Å. As a result, the large interface energy limits nucleation on the bare Si substrate, and the aftermath is the poor vertical alignment of ZNRs on Si. The purpose of the seeds is to lower the interfacial energy at the ZnO–Si interface and provide required nucleation sites for the axial growth of ZNRs via heterogeneous nucleation. Hence, P–Si was used as a test sample for the growth of nanostructures. We tested the growth of ZnO nanostructures on a seeded glass substrate as well which was totally harmonious to the microwave environment.

**Figure 2:**
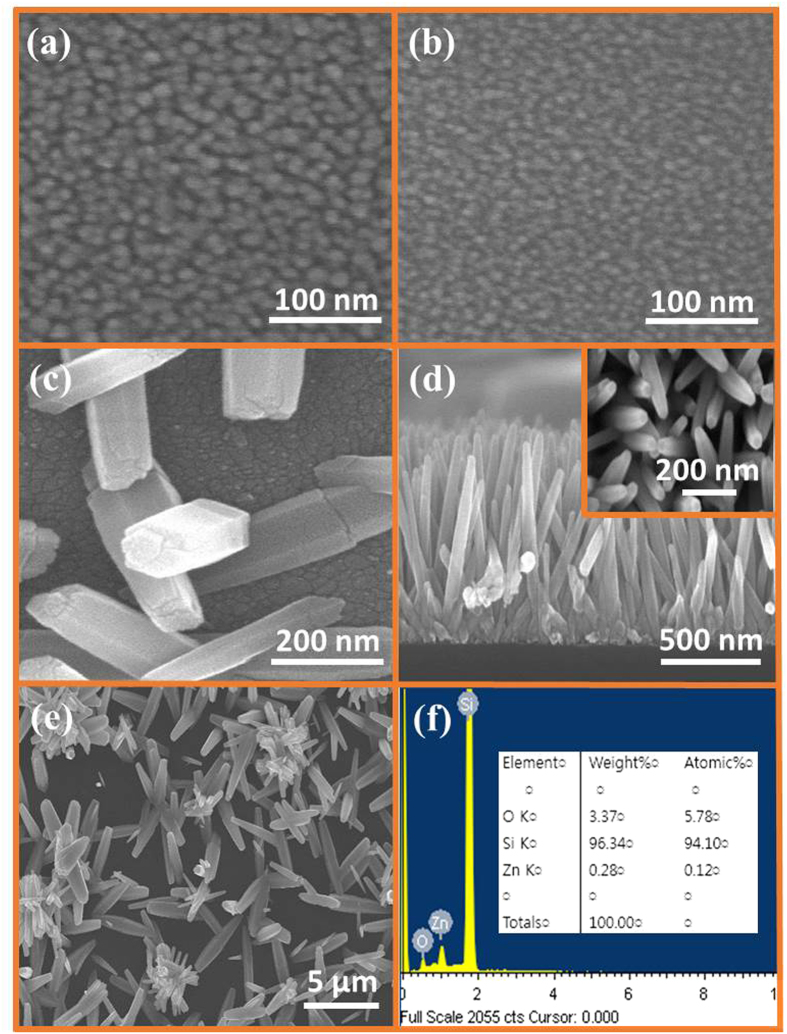
SEM images of (**a**) five times coated seeds, (**b**) twice coated seeds, (**c**) ZNRs grown on high concentration (0.044 M) seeds with the glimpse of seeds in the backdrop, (**d**) ZNRs running into one another because of the space confinement effect with the corresponding top view in the inset, (**e**) ZNRs grown on unseeded substrate, and (**f**) EDS of thin film ZnO seeds with the atomic ratio of the elements.

Figure 2(a,b) show the SEM images of a thin film of ZnO seeds coated on a P–Si substrate five times and twice, respectively. The thickness and diameter of the seeds depend on the number of seed layer coatings and the seed solution concentration, as can be seen in the figure as the twice-coated seeds have a small diameter than the five-times-coated seeds. Figure 2(c) depicts the image of ZNRs grown in a high concentration seed solution (0.044 M) coated on the substrate just twice. It is evident that the seeds have a quite large diameter and the grown ZNRs are scanty, laid down, and have very large dimensions. Hence, the diameter, thickness, and density of the seeds depend on the concentration of the seed solution and the number of seed layer coatings. In addition, the larger the diameter of the seed, the larger the diameter of the ZNRs.

Increasing the seed layer thickness increases the nanowire density, improves the degree of vertical alignment, and sharply decreases the length. This is because of the space confinement effect of the densely packed ZNRs where a few of the ZNRs run into their neighboring ZNRs impeding the growth, as shown in Fig. 2(d), with the inset revealing tilted ZNRs in the top view. The tallest ZNRs are those that grew perpendicular to the substrate; hence, for seed concentration and thickness, we have to find an optimum point at which the growth of the ZNRs gives the best possible value. In our system, we found it to be 0.022 M zinc acetate solution coated five times on the substrate. Additionally, ZNRs grown on an unseeded substrate had quite large dimensions, as shown in Fig. 2(e), but all of them were laid down because of the absence of seeds. Furthermore, the EDS data in Fig. 2(f) elucidates the purity of the seeds that are only composed of Zinc and Oxygen elements. A large peak for Si in the EDS divulges that the seed layer is quite thin and that P–Si is used as a substrate.

### Microwave-assisted direct growth of ZNRs

**Figure 3:**
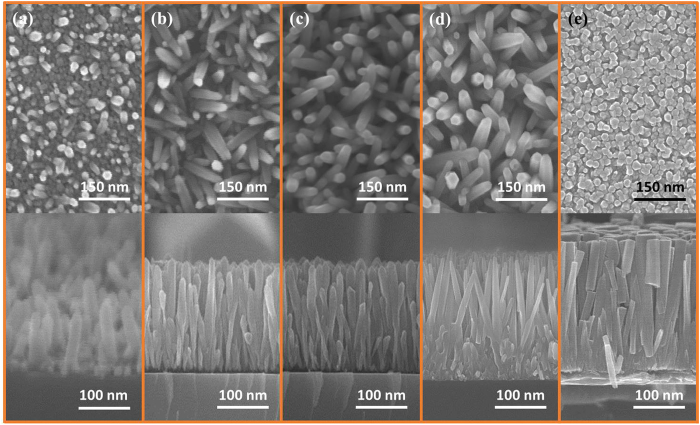
Top and cross-sectional SEM images of ZNRs grown for (**a**) 2 mins, (**b**) 5 mins, (**c**) 7 mins, (**d**) 15 mins, and (**e**) ZNRs start fusing into one another to form a film-like structure.

Figure 3 illustrates the SEM images of ZNRs grown on a P–Si substrate via a microwave-assisted hydrothermal method in 25 mM solution placed in a domestic microwave oven. It could be seen in Fig. 3(a) that the nucleation begins after only 2 mins, which is quite fast, but ZNRs are not fully grown with the glimpse of the seeds that are also visible on the base. With the passage of time, these nascent ZNRs continue growing as shown in Fig. 3(b–d), which were taken after 5 mins, 7 mins, and 15 mins growth time, respectively. The length and diameter of the ZNRs were almost the same for 5 mins and 7 mins growth time. Furthermore, it is explored that by increasing the molar concentration of the solution, the diameter and length of the ZNRs also increased linearly, but the aspect ratio remained the same. Eventually, the diameter of ZNRs will continue increasing for higher concentration solutions until the point at which the ZNRs start fusing into one another to form a film-like structure, as illustrated in Fig. 3(e).

**Figure 4:**
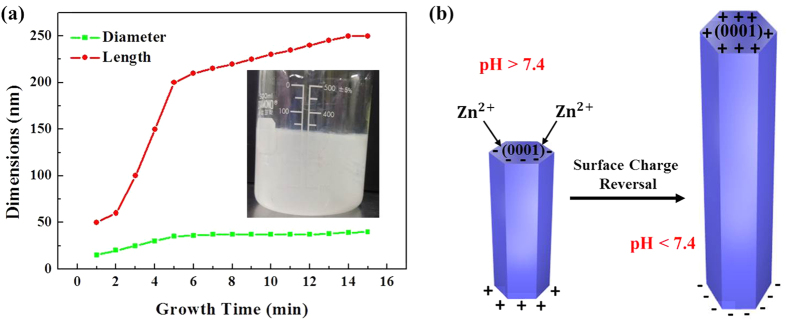
(**a**) Diameter and length of ZNRs vs. growth time. The inset shows the homogeneously nucleated solution at 5 mins, and (**b**) Schematics of the pH-dependent growth of ZNRs.

It is found that the length and diameter of the ZNRs increased until 5 mins of irradiation, where their values saturate, as portrayed in Fig. 4(a). If the time is further increased up to 15 mins, the dimensions and morphology of the ZNRs remain almost unchanged. The growth stoppage point is monitored at around 5 mins, after which the values of ZNR’s length and diameter almost saturate because of the ultrafast supersaturation of zinc salts. To further qualify the value of growth stoppage, we recorded the change in the color of the solution prior to irradiation and after 5 mins of irradiation. The inset in Fig. 4(a) shows that the solution’s color changes from transparent prior to irradiation to a dark milky color as the growth time reaches 5 mins. The color change is due to the formation of ZnO nanostructures inside the solution (homogeneous nucleation) rather than on the seeded substrate (heterogeneous nucleation). The low interfacial energy at ZnO–Si interface prefers heterogeneous nucleation but still, at very high temperatures under high power microwave irradiations, some of the reactants are homogeneously nucleated inside the solution rather than on the seeded substrate. The homogeneously nucleated growth material is wasted which results in growth stoppage of the nanostructure because no more reactants are available for the growth of nanostructures on a seeded substrate. Hence, it is found in our method that 5 mins is the transition time at which homogeneous nucleation supersedes heterogeneous nucleation. Therefore, besides having a very short growth time, one common drawback of direct heating is the ultra-fast depletion of the growth solution, which ultimately results in growth stoppage. However, the method has its own applications where nano-scaled ZNRs are needed and time is restricted to 5 mins, but to deal with the issue, we performed different successful experiments with quite stunning results as suggested in the next sections.

### ZNRs growth mechanism under microwaves

The chemical reactions inside the solution in the presence of microwaves are:


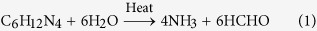



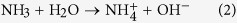



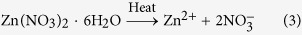



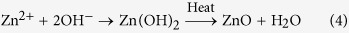


Methenamine is a nonionic cyclic tertiary amine whose role is quite complex and still under debate. In our case, its role is to coordinate to the ZnO crystal, hinder the growth of certain surfaces for the axial growth of ZNRs, and to act as a pH buffer in the solution. The decomposition of one methenamine molecule provides four NH_3_ molecules and six HCHO molecules (eq. (1)), where the HCHO molecules do not take part in the rod growth. The OH^−^ ions are produced via the protonation of NH_3_ (eq. (2)) and the Zn^2+^ ions are released by Zn(NO_3_)_2_·6H_2_O in water (eq. (3)) which reacts with OH^−^ to form Zn(OH)_2_ which is further decomposed under microwave-assisted heat to form ZnO nanostructures (eq. (4)). The pH of the solution, which is another vital factor in controlling the growth, was naturally maintained from 6 to 9 without additional chemicals. The measured values of pH of the solution before, during, and after the reactions are 6.9, 7.8, and 6.8, respectively. The shift in the pH values was quite rapid and happened in the span of just 5 mins in microwave environment.

The growth mechanism of ZNRs is closely related to the surface charge at the hexagonal surface, which ultimately depends on the pH value of the growth solution as elaborated in Fig. 4(b). The isoelectric point of the methenamine-assisted hydrothermally grown ZnO is 7.4^32^. Because of the provision of the stream of OH^−^ ions via the decomposition of methenamine (eq. (1) and eq. (2)), the local pH of the solution goes beyond 7.4 and the surface charge becomes negative. The top 0001 negative surface, which is the most energetic surface for nucleation and growth, attracts Zn^2+^ ions for the growth of ZnO along the c-axis. With the depletion of OH^−^, the pH falls below 7.4, which is its isoelectric point, and the polar bases on ZNRs are reversed to Zn^2+^-terminated 0001 positive and O^2−^-terminated 000ī negative. The allocation of a positive charge on the growth front is ominous for the further growth of ZNRs, as the positive 0001 surface restricts the attraction of further Zn^2+^ ions and the phenomenon is pre-eminent as growth stoppage. The remaining six low-indexed, non-polar, and most stable planes, denoted as (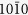), are also grown parallel to the c-axis. The low surface energy in the non-polar and high surface energy in the polar planes make them respectively stable and unstable, and help grow ZNRs vertically. For a ZnO crystal, the growth rate of multiple facets is marked as 0001 > 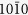 > 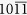 > 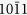 > 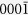.

### Microwave-assisted solution-replacement method

**Figure 5:**
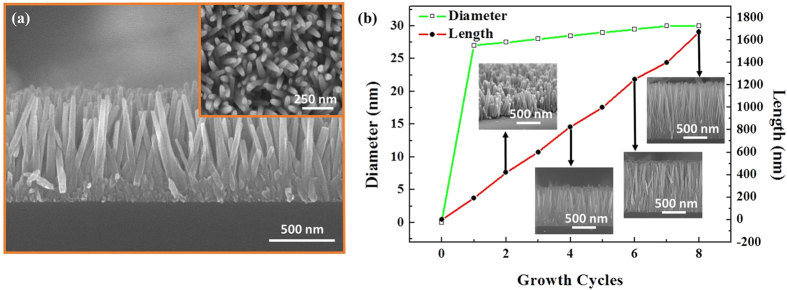
(**a**) Cross-sectional SEM image of ZNRs grown when replacing the solution four times, with its corresponding top view in the inset, and (**b**) Behavior of length and diameter of the ZNRs vs. the growth cycles. The insets reveal the cross-section of ZNRs at the corresponding growth cycle.

The method is introduced to address the problem of growth stoppage in a direct heating method. The basic analogy of the method is to replace the depleted solution with fresh solution after the specified interval so that new material becomes available for the vertical growth of ZNRs. Figure 5(a) shows a cross-sectional SEM image, with the top view in the inset, of ZNRs grown for four five-minute cycles of 25 mM growth solution. It is inferred from the cross-sectional SEM image that although the cumulative growth time is also increased, the fresh stream of reaction solution provides sufficient reactant to grow ZNRs vertically by up to 800 nm. Figure 5(b) shows the length and diameter of the ZNRs as a function of the growth cycles. The insets show the corresponding cross-sections of ZNRs at particular growth cycles. The lengths of ZNRs increase linearly with the average growth rate of 200 nm per five-minute cycle, which is also in concurrence with the experiment performed in the section of the microwave-assisted direct growth method. The length continues to increase as the number of cycles increases, but the value of the diameter saturates at approximately 30 nm. Initially, when the premature ZNRs nucleate from the seeds, the radial growth rate of a ZNR is higher until the formation and establishment of the lateral walls. With the passage of time, stable lateral walls halt the radial growth and increase the axial growth rate of ZNRs. When a sample with already-grown ZNRs is introduced in a fresh solution, the ZNRs are already mature with quite strong and stable lateral walls, which only allow it to grow axially rather than radially. Second, only the polar top and bottom surfaces of the ZNRs attract Zn^2+^ and O^2−^ ions, respectively. Hence, most of the reactant solution serves the axial growth, which increases the aspect ratio.

### Microwave-assisted preheating method

**Figure 6:**
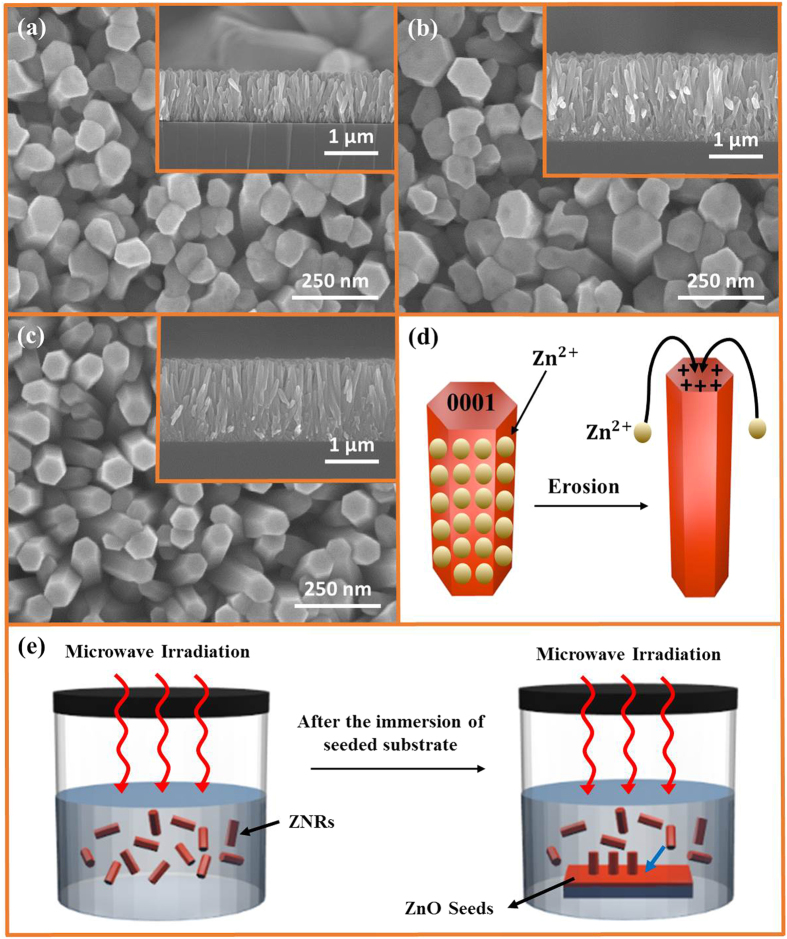
SEM images of ZNRs grown with preheating method for (**a**) 5 mins preheating, (**b**) 10 mins preheating, (**c**) 15 mins preheating, (**d**) Schematics of the erosion and deposition of Zn^2+^ in the 15 mins preheating method, and (**e**) Schematics of the ZNRs formation and deposition phenomena in the preheating process.

The preheating hydrothermal method was first introduced by Ashfold and his coworkers^33^. It took 10–60 hours for Ashfold to grow ZNRs and ZnO nanotubes. In this experiment, we present the ultrafast growth of ZNRs via a microwave-assisted preheating hydrothermal method in just 20–30 mins. Our method is not only ultrafast for the growth of ZNRs, it also produces quite long ZNRs compared to the only 400 nm long ZNRs created by Ashfold. The effect of preheating time on the dimensions of ZNRs is broached in this segment. As shown in Fig. 6(a), the ZNRs grown with 5 mins of preheating are erected perpendicular to the substrate and densely populated. The diameters of the ZNRs are in the range 95–105 nm with an average diameter of 100 nm and an average length of approximately 900 nm. The shapes of the ZNRs were not perfectly hexagonal; some of the ZNRs were tilted towards one another and made ZnO clusters, which added to the deformation of their shape. The formation of these clusters is due to the interfacial surface energy and capillarity between the adjoining ZNRs, which allows them to tilt and merge into one another.

Both the length and diameter of the ZNRs can be increased by increasing the preheating time to 10 mins, as shown in Fig. 6(b). The ZNRs are again closely packed, densely populated, and merged into one another making clusters. Again, the shapes of the ZNRs are not perfectly hexagonal. One interesting phenomenon found when using 10 mins preheating is the formation of a hole at the top of the ZNRs, as seen in the top view of Fig. 6(b). The diameter of the hole is approximately 20–30 nm, and it is present in almost all ZNRs. The erosion at the top polar and unstable surface during the long preheating time might be the reason for this hole formation. The 10 mins–preheated ZNRs are quite long and grown vertically perpendicular to the substrate compared to the 5 mins preheated ZNRs, as manifested in the insets of the cross-sectional view (see Fig. 6(a,b)).

Figure 6(c) shows the grown ZNRs when the preheating time was further raised to 15 mins. It is epitomized that the solution depletes after 10 mins preheating, as the length of the ZNRs after 10 mins and 15 mins preheating remains the same. The long preheating time has amazingly reduced the diameter of the ZNRs to approximately 70 nm. Furthermore, the pores/holes, produced at the top of ZNRs in the 10 mins preheating method are refilled, and the shapes of the ZNRs are perfectly hexagonal. The phenomenon responsible for the production of this hexagonal shape, filling the holes, and the reduction of the ZNRs diameters in such a high concentration solution (0.1 M) can be attributed to the dissolution, transport, and regrowth of Zn^2+^ ions from side walls to the top surface of the ZNRs. The further increase in the preheating time to 15 mins shifts the chemical reaction in eq. (4) to the left, where ZnO is being dissolved and the Zn^2+^ ions are formed under such a high temperature and long preheating time. Generally, as per the kinetics, the dissolution rate of unstable 0001 ZNR polar faces is faster than that of the ultrastable 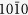 nonpolar walls. In our case, the 0001 face may also dissolve as shown by the formation of holes in Fig. 6(b), but the dissolution rate of the 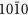 walls was faster than the 0001 top surface, because of the quite large surface area availability compared to the small diameter of ZNRs under continuous exposure to high power microwaves. The produced Zn^2+^ ions in the reverse reaction are transported to the zinc-terminated top polar surface of ZNRs that, besides reducing the diameter, increase the length and aspect ratio of the ZNRs by only a few nm. The whole process is demystified in Fig. 6(d). The process both fills the holes in the ZNRs and revamps the shape of ZNRs into a perfect hexagon. Hence, the experiment establishes that the preheating time is crucial for the growth of ZNR arrays.

Figure 6(e) depicts the schematics of the whole fabrication process such as long ZNRs in a short time via a preheating method. The basic analogy is to keep a check on two antithetical processes named homogeneous and heterogeneous nucleations. According to eq. (1)–(4), 2, 3, 4, the reactants are allowed to react in the solution under high-intensity microwaves. Without the presence of a seeded substrate, the only process going on inside the solution is homogeneous nucleation. The color of the solution turns milky during preheating, which is an indication of the formation of ZNRs inside the solution. The moment a seeded substrate is immersed inside the solution, the already-formed ZNRs are instantly transferred to the seeded sites because of the presence of lower interfacial energy at the ZNR–ZnO seed layer interface. The length of the ZNRs, transferred to the seeded substrate, is already quite long due to the absence of heterogeneous nucleation during preheating. After immersion of seeded substrate inside the solution, the length only increases further because the only process supporting the growth is now heterogeneous nucleation. There is no room for homogeneous nucleation during the post substrate immersion growth time, because the reaction solution is already depleted during preheating, which only allows the growth of high-aspect-ratio ZNRs on the seeded substrate.

### PEI-based microwave-assisted growth method

**Figure 7:**
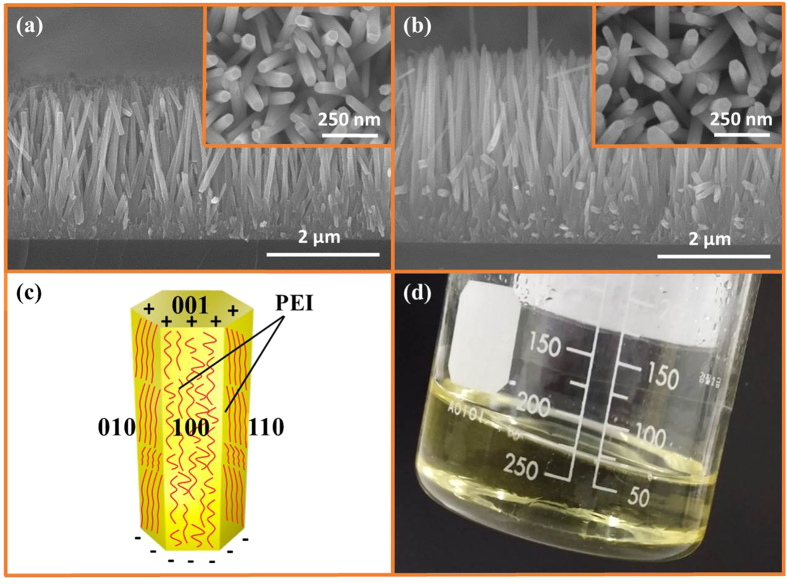
(**a**) Cross-section and top view in the inset of ZNRs grown with PEI in 15 mins, (**b**) Cross-section and top view in the inset, of ZNRs grown with PEI in 25 mins, (**c**) PEI circumambulated around the ZNR, and (**d**) Color of the solution, which confirms the Mannich reaction between zinc salts and PEI.

Another method of addressing the issue of rapid growth stoppage in a microwave oven is the use of surfactants. PEI is a nonpolar polymer that has recently been reported as being used as a surfactant to increase the aspect ratio of the solution-grown ZNRs. But the orthodox PEI-based hydrothermal methods take more than 15 hours to grow ZNRs with moderate aspect ratio. In this experiment, high-aspect-ratio and high-quality ZNRs are grown in the span of just 15–25 mins in a microwave oven. Figures 7(a,b) show SEM images of ZNRs grown with 2–5 mM PEI in 25 mM solution for 15 and 25 mins, respectively. The inset reveals corresponding top views of the images. The lengths of the ZNRs are interestingly long and the diameters are restricted to 60–70 nm, increasing the aspect ratio of the ZNRs. The ZNRs are aligned perpendicular to the substrate, perfectly hexagonal, and densely populated. Because of the strong chelating ability of PEI, the diameter of the ZNRs remains quite low at 60–70 nm, while the length increases from 2.3 μm to 3.2 μm at 15 mins and 25 mins, respectively. As a surfactant, PEI contains a large number of amino groups in a long molecular chain that can be radially protonated between the pH values of 3 and 11. The protonated amino groups modify the surface free energy and the growth velocity of the ZNRs by adsorbing on the non-polar lateral walls of the ZNRs because of the electrostatic affinity^34^, as depicted in Fig. 7(c). Therefore, the growth of ZNRs, circumambulated with PEI, is impeded in the radial 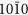 direction and the growth in axial 0001 direction is aggrandized, which results in the growth of high-aspect-ratio ZNRs. It also establishes that the method is no more supportive to the growth stoppage problem as the length continues to increase with the passage of time from 15 to 25 mins. In addition, the color of the solution did not change to a dark milky color, but was changed to a pale yellow, as revealed in the photograph of the solution in Fig. 7(d) taken after 25 mins, which reveals the absence of homogeneous nucleation even in the presence of ultrafast supersaturation during growth. The yellow color of the solution is because of the Mannich reaction between the PEI and the compound produced after the decomposition of methenamine^35^. All reactants are used for the growth of ZNRs, which helps increase the length of the ZNRs. The most important factor is again the ultrafast fabrication of high-aspect-ratio ZNRs in just 15 to 25 mins, which would take hours otherwise.

The fact that the high concentration PEI solution may decelerate the growth of ZNRs is by virtue of the increased chelating ability of PEI to Zn^2+^, which halts the formation of Zn(OH)_4_^2−^ growth units. It also decreases the decomposition rate of methenamine, because of the improved pH at high concentrations. Hence, increasing the PEI concentration beyond 5 mM could suppress the ZNR growth completely, and it could go further to erode the ZnO seeds away from the substrate. The solution to the problem is to use PEI within the stipulated units, 2–5 mM, and to preheat the solution prior to the immersion of the seeded substrate into the solution, so that a few of the methenamine molecules decompose beforehand. Furthermore, as demonstrated in the seed layer section above, thin seeds foster small diameter ZNRs. Hence, we believe that seed layer thinning because of the low degree of supersaturation under the influence of PEI is another reason for the growth of small-diameter high-aspect-ratio ZNRs in this method.

### Microwave-assisted growth of ZnO nanowalls and nanoflowers

**Figure 8:**
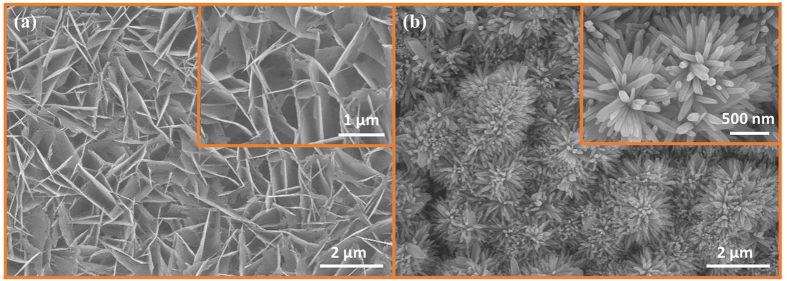
SEM images of (**a**) ZNWs and (**b**) ZNFs with each corresponding high magnification image in the inset.

Figure 8(a) shows the morphology of fabricated ZNWs. The ZNWs are vertically well-aligned and all the walls are connected to one another as can be seen in the magnified image in the inset. Every segment of the wall has a different length, but the average length of a single segment of ZNW is approximately 1 μm and the width is only 10–20 nm. It is believed that the formation of ZNWs is due to defect-selective dissolution processes^36^. The OH^−^ ions that are required to selectively etch ZNRs away at the polar 0001 surface are provided by NH_4_OH in the solution at 60–70 °C. The chemical equation for the dissolution of nascent ZnO complexes is:


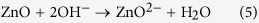


Hence, the factors necessary for the fabrication of ZNWs are: 1) A high concentration of seeds, 2) a high concentration (50–75 mM) of precursor solution, 3) the presence of excessive OH^−^ ions in the solution, and 4) 60–70 °C under 2 mins of microwave irradiation. As per our knowledge, the microwave-assisted growth of ZNWs in just 2 mins is one of the fastest fabrication techniques propounded until now.

Figure 8(b) illustrates the SEM image of ZNFs with the magnified image in the inset. The ZNFs are in an ideally perfect shape and are closely packed. The length of each petal of the flower is approximately 1 μm with a diameter of 40 nm. All the petals of the ZNFs originate from a single stem and form an impeccable flower-like structure. Traditionally, at pH values of around 7, ZnO is formed by the reaction explained in eq. (4). With the addition of NH_4_OH as a mineralizer, the pH of the solution is elevated to 12, and there are more OH^−^ ions available for growth than Zn^2+^ ions at this particular juncture. Hence, rather than the growth of ZNRs in eq. (4), the exorbitant OH^−^ ions allow the following reaction to follow immediately^37^.


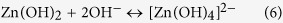


At temperatures above 100 °C and high pH, the [Zn(OH)_4_]^2−^, obtained in eq. (6), is believed to be a new growth species and the ZnO nuclei formed in eq. (4) serve the purpose of seeds. Now, the growth of ZNFs depends upon the number of active sites that are available on the seed and the number of growth species surrounding those sites. In the presence of a plethora of OH^−^ ions, the number of growth species increases manifold and are attached on all the nuclei to form a flower-like structure as shown in Fig. 8(b). Hence, it is believed that high temperature, under high power microwave irradiation, and high pH value are central factors to influencing the morphology of ZnO nanostructures. Furthermore, the ultrafast growth of such a refined ZNF structures, in just 5–10 mins, is propounded for the first time in a microwave environment.

### XRD/EDS/PL data analysis

**Figure 9:**
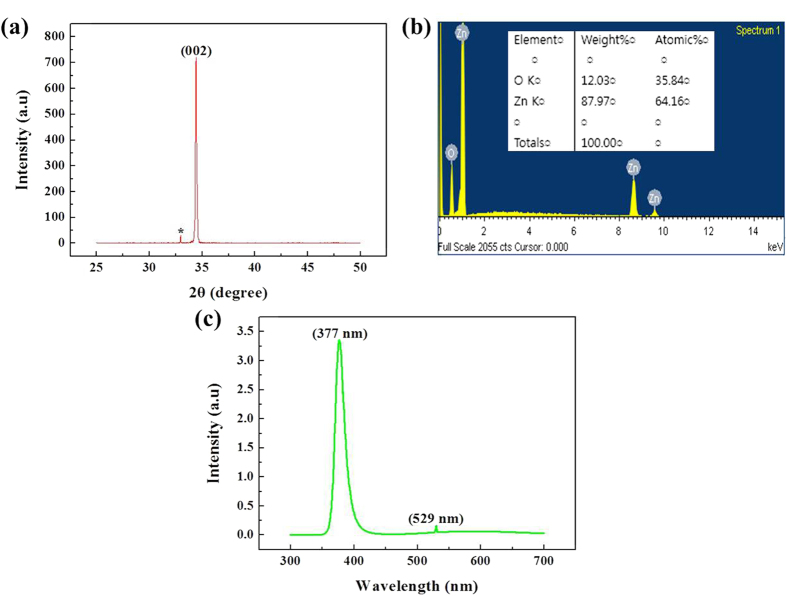
(**a**) XRD, (**b**) EDS, and (**c**) PL spectra of ZNRs grown with the PEI-based method.

XRD patterns of ZNRs were taken to examine the crystal structure of ZNRs from the microwave-assisted PEI-based growth method. XRD, EDS, and PL data collected from other methods, i.e., microwave-assisted direct growth, solution-replacement, and preheating methods, are also given in supplementary information (see Figs S1, S2 and S3). It could be seen in Fig. 9(a) that only a single peak at 34.4° is observed that could be indexed as 002 of the wurtzite structure of ZnO. It shows that the grown ZNRs are not only highly crystalline, but are also excellently oriented along the c-axis direction, which is also in concurrence with the SEM data. In contrast to the XRD spectra of ZNRs prepared with an orthodox hydrothermal method wherein multiple XRD peaks are observed, our method propounds ZNRs with better orientation and purity. Furthermore, a much higher and narrower full-width-half-maximum peak at 002 is comparable to the ZNRs prepared with MOCVD or VLS methods. A small kink indexed with an asterisk (*) is supposed to be a response from a Si substrate. Additionally, the EDS spectrum in Fig. 9(b), with the atomic ratio in the inset also confirms the purity of ZNRs where only zinc and oxygen are present at an almost stoichiometric level. Figure 9(c) shows the room-temperature PL spectra of grown ZNRs. The spectra primarily consist of two emission bands: A strong emission band at ~377 nm (3.3 eV) and an unusual very weak green band kink at ~529 nm (2.35 eV). The strong UV emission corresponds to the band-to-band excitonic recombination related to the near-band-edge emission of ZnO. The point to ponder is the absence of a broad visible spectrum peak. The low-intensity green emission kink is due to the presence of only a few oxygen vacancies in the ZnO crystal imbibed during the preparation of the sample^38^. The absence of any other high-intensity peaks and the presence of a very high-intensity UV peak reveals that the grown ZNRs are highly pure with perfect crystallinity^39^.

## Conclusion

In this study, the ultrafast and facile growth of ZnO nanostructures in a domestic microwave oven has been presented and multiple methods to address the problem of growth stoppage have been explored. A microwave-assisted direct heating method is used to examine the basic growth procedure and to probe the effect of solution concentration and growth time on the growth of ZNRs. Furthermore, a growth stoppage point is found by dint of the direct heating method. The problem of ultrafast growth stoppage has been addressed via microwave-assisted solution replacement, preheating, and PEI-based growth methods. It has been testified that by replacing the solution method that the aspect ratio of the ZNRs is directly proportional to the number of growth cycles used to grow ZNRs. The preheating method has a quite complex chemistry, but it is possible to grow high-aspect-ratio ZNRs where the length and the diameter of the ZNRs depend upon the preheating time. In the last method, PEI has been utilized as a surfactant and chelating agent for the fabrication of quite long ZNRs. The growth of high-quality ZNWs and ZNFs have also been presented in just 2 and 10 mins, respectively. It is found in XRD, EDS, and PL measurements that the grown nanostructures are highly pure with a high degree of crystallinity. All methods have been critically analyzed and their pros and cons have been presented. The potential applications of ultrapure ZnO nanostructures fabricated in a short time are in the realm of optoelectronics, lasing, sensors, transistors, and solar cells. All the presented methods could be applied to the fabrication of other semiconductor materials via hydrothermal and solvothermal methods.

## Methods

### Materials preparation

All chemicals were commercially available and were of analytical grade, and hence were used without any further purification. Furthermore, ultra clean DI water of 18.2 MΩ was used where necessary. P–Si (crystal orientation of 100 and resistivity of 1–10 Ωcm) was used as a substrate in all experiments. Zinc acetate dihydrate [Zn(CH_3_COO)_2_·2H_2_O] (MW 219.51 g/mol) and n-propanol [C_3_H_8_O] (MW 60.10 g/mol) were used for the thin film deposition of ZnO seeds, and zinc nitride hexahydrate [Zn(NO_3_)_2_·6H_2_O] (MW 297.48 g/mol) and methenamine [C_6_H_12_N_4_] (MW 140.186 g/mol) were mobilized to create an aqueous precursor solution with specified molarity. ZNRs were grown inside a commercial microwave oven with a Teflon sample holder immersed in a 500 ml glass beaker. Polyethylenimine (PEI) (end-capped, MW 800 g/mol LS) was used as a chelating agent for the growth of long ZNRs and ammonium hydroxide [NH_4_OH](MW 35.05 g/mol, 28% NH_3_ in H_2_O) was used for the grown of ZNWs and ZNFs.

### ZnO seed layer formation

The P–Si substrate was first cleaved into 1 × 1 inch segments and then washed with BOE and DI water and dried with nitrogen gas to etch away the formed oxide layer on the surface of P–Si. zinc acetate dihydrate (0.022 M) was dissolved in 10 ml n-propanol and sonicated to the point at which the color of the solution changed from transparent to milky white, then back to transparent. The observation of the solution’s color change is imperative when making a saturated solution. The seeds were dropped and spin coated at 3000 rpm on the substrate for 30 seconds, and the process was repeated for a number of times that depended on the thickness of the required thin film. For the first four coatings, the sample was annealed at 110 °C, and for the last coating, it was annealed at 310 °C for 30 mins so that the adhesive and sticky nucleation layer of the seeds was formed on the surface of the substrate^40^.

### Experimental constraints of ZnO nanorod growth

For the growth of ZNRs, a precursor solution was made by mixing equimolar zinc nitrate hexahydrate and methenamine in DI water. After one hour of continuous stirring, an aqueous solution of both chemicals was ready to be used for the growth of ZNRs. After this, the seeded and unseeded substrates were immersed inside the solution beaker and placed inside the microwave oven where the solution with the immersed samples was exposed to 2.45 GHz microwave radiation. The beaker revolved inside the oven at 4 rev/min, which ensured the homogenous transmission of heat to the solution. The *in situ* temperature monitoring of the solution was also performed via a wired thermometer, and the temperature was raised at 25 °C/min. The solution reached supersaturation after only 4 mins, which might boast precursor dissolution and ultimately hamper the growth of ZNRs. To circumvent the ultrafast supersaturation of the solution and control the reaction temperature, a pulsed microwave irradiation method was used in which the exposure of microwaves to the setup was cut-off for 30 seconds after every 60 seconds exposure. It is difficult to control the exposure of microwave irradiations to the solution inside the microwave oven. Thence, in our experiment, it is actually the growth time that is the most vital factor to control the temperature of the solution to a certain degree. To keep a check on the homogeneous and heterogeneous nucleation processes inside the solution, we performed four different experiments to determine effective routes for increasing the length of the ZNRs with their corresponding pros and cons; these methods are as explained below.

### Microwave-assisted direct heating method

This method was exploited to examine the effect of different molar concentrations and growth times on the diameter and length of ZNRs. Most importantly, the inquisition of the point where the growth solution depleted in favor of homogeneous nucleation is realized with this method. In our investigation, 25–75 mM of zinc nitrate hexahydrate and methenamine were dissolved in DI water and the morphological effects of the molar concentration on the ZNRs were recorded. ZNRs were grown for 2–15 mins to study the evolution of the morphology of ZNRs as a function of the growth time and to determine the optimum point at which ZNR growth is saturated. To further validate our claim, the apparent change in the color of the reaction solution was also monitored at different growth times.

### Microwave-assisted solution-replacement method

The solution was prepared by mixing equimolar (25 mM) zinc nitrate hexahydrate and methenamine in DI water. The seeded and unseeded substrates were immersed inside the solution and exposed to microwave radiation. The substrates were then removed from the solution after every 5 mins of radiation, washed with DI water, and reimmersed in a fresh solution of the same molarity. The procedure was repeated 2–8 times. Finally, the substrates were rinsed in DI water and dried with nitrogen gas blow.

### Microwave-assisted preheating method

Equimolar zinc nitrate hexahydrate and methenamine were mixed to form a 0.1 M solution in DI water. The solution was preheated in a microwave oven by exposing the solution to microwave radiation for 5 mins, 10 mins, and 15 mins. Afterward, the seeded substrates were immediately immersed in all the aforementioned preheated solutions and the solutions were again exposed to microwave radiation for a fixed post preheating interval of 15 mins. Then, the as-grown samples were taken out, rinsed in DI water, and dried with nitrogen. The ramifications of the preheating time on the morphology, composition, crystallinity, and optical properties of the ZNRs were probed.

### PEI-based microwave-assisted growth method

The solution was concocted by dissolving 25 mM zinc nitrate hexahydrate, 25 mM methenamine, and 2–5 mM PEI in DI water and continuously stirring. After one hour, the solution was preheated for 2–3 mins, and then the seeded substrates were immediately transferred to the solution, which was then exposed to microwave radiation for 15–25 mins. Once the solution changed color from transparent to dark yellow, the substrates were removed from the solution and rinsed with DI water. The effects of PEI concentration and growth time on the morphology, composition, crystallinity, and optical properties of the ZNRs were studied.

### Ultrafast fabrication of ZnO nanowalls and ZnO nanoflowers

The solution was prepared by dissolving 50–75 mM zinc nitrate hexahydrate and 50–75 mM methenamine in DI water. To step-up the solution’s pH, ammonium hydroxide [NH_4_OH] was used as a mineralizer, where 15–20 ml of it was added to the solution, which was then set to stirring. After one hour of continuous stirring, the seeded P–Si substrates were immersed in the solution and the solution was exposed to microwave radiation. The respective growth times for ZNWs and ZNFs were just 2 mins and 10–15 mins. After growth, the solution was allowed to cool to room temperature, and then the samples were taken out, rinsed in DI water, and dried with nitrogen gas.

### Characterization tools

The morphological structure and the elemental composition of the grown ZnO nanostructures were measured with scanning electron microscopy (SEM: Hitachi S-4800) and energy dispersive X-ray (EDS) analysis attached with the same system. The crystalline nanostructures were recorded with X-ray crystallography (XRD: Rigaku Ultima IV) and the optical properties were measured with photoluminescence (PL: Accent RPM 2000) spectroscopy. The pH and temperature of the solution were recorded with digital pH and thermometer, respectively, and the solution colors were also monitored.

## Additional Information

**How to cite this article**: Rana, A. H. S. *et al.* Microwave-assisted Facile and Ultrafast Growth of ZnO Nanostructures and Proposition of Alternative Microwave-assisted Methods to Address Growth Stoppage. *Sci. Rep.* **6**, 24870; doi: 10.1038/srep24870 (2016).

## Supplementary Material

Supplementary Information
