# Supplementary material for: Microwave-assisted Facile and Ultrafast Growth of ZnO Nanostructures and Proposition of Alternative Microwave-assisted Methods to Address Growth Stoppage
Source: Sci Rep. 2016 Apr 22;6:24870. doi: 10.1038/srep24870 (PMC4840357; doi:10.1038/srep24870)
Supplement: Supplementary Information [file srep24870-s1.pdf]

Microwave-assisted Facile and Ultrafast Growth of ZnO Nanostructures and Proposition of Alternative Microwave-assisted Methods to Address Growth Stoppage

Abu ul Hassan Sarwar Rana, Mingi Kang, and Hyun-Seok Kim*

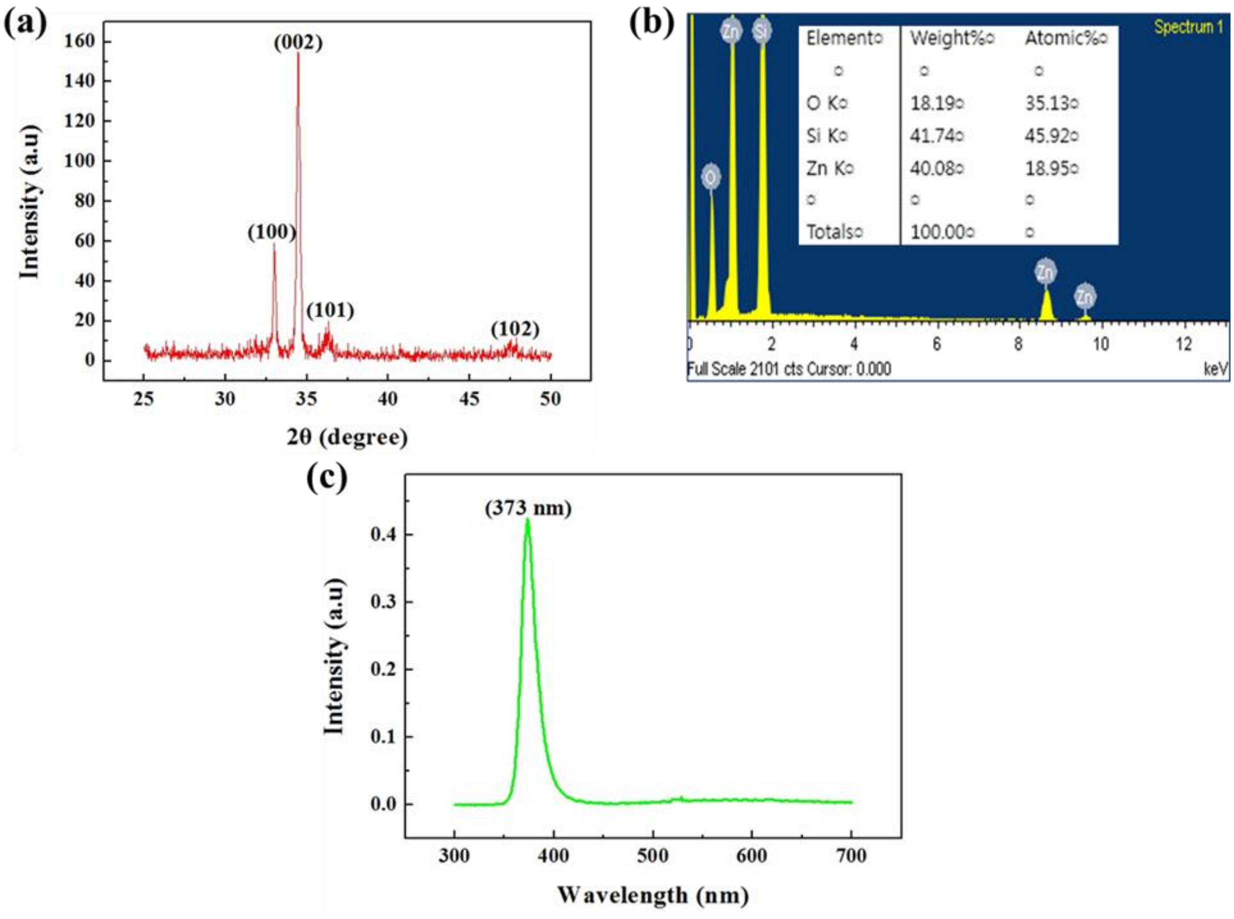

Figure S1. (a) XRD, (b) EDS, and (c) PL spectra of ZNRs grown with the microwave-assisted direct growth method.

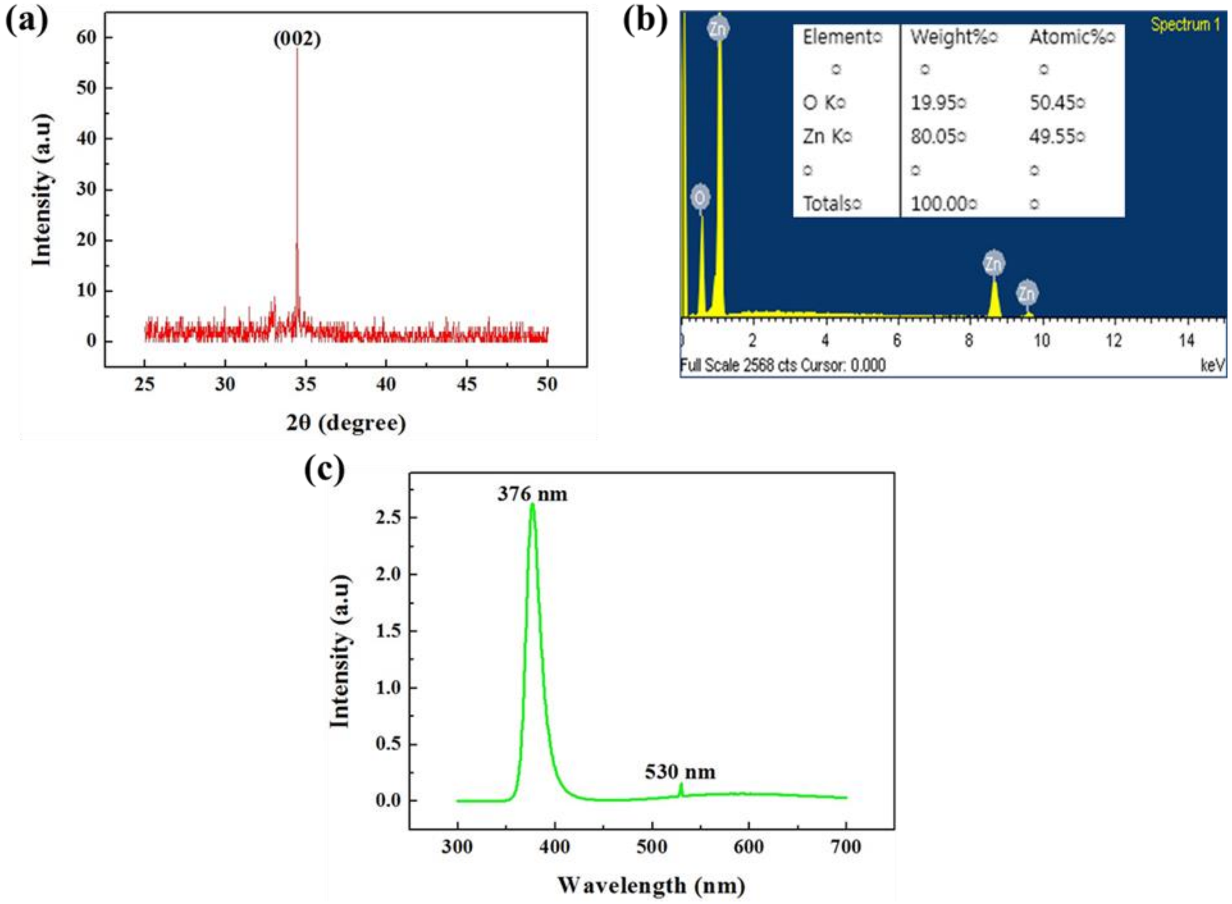

Figure S2. (a) XRD, (b) EDS, and (c) PL spectra of ZNRs grown with the microwave-assisted solution-replacement method.

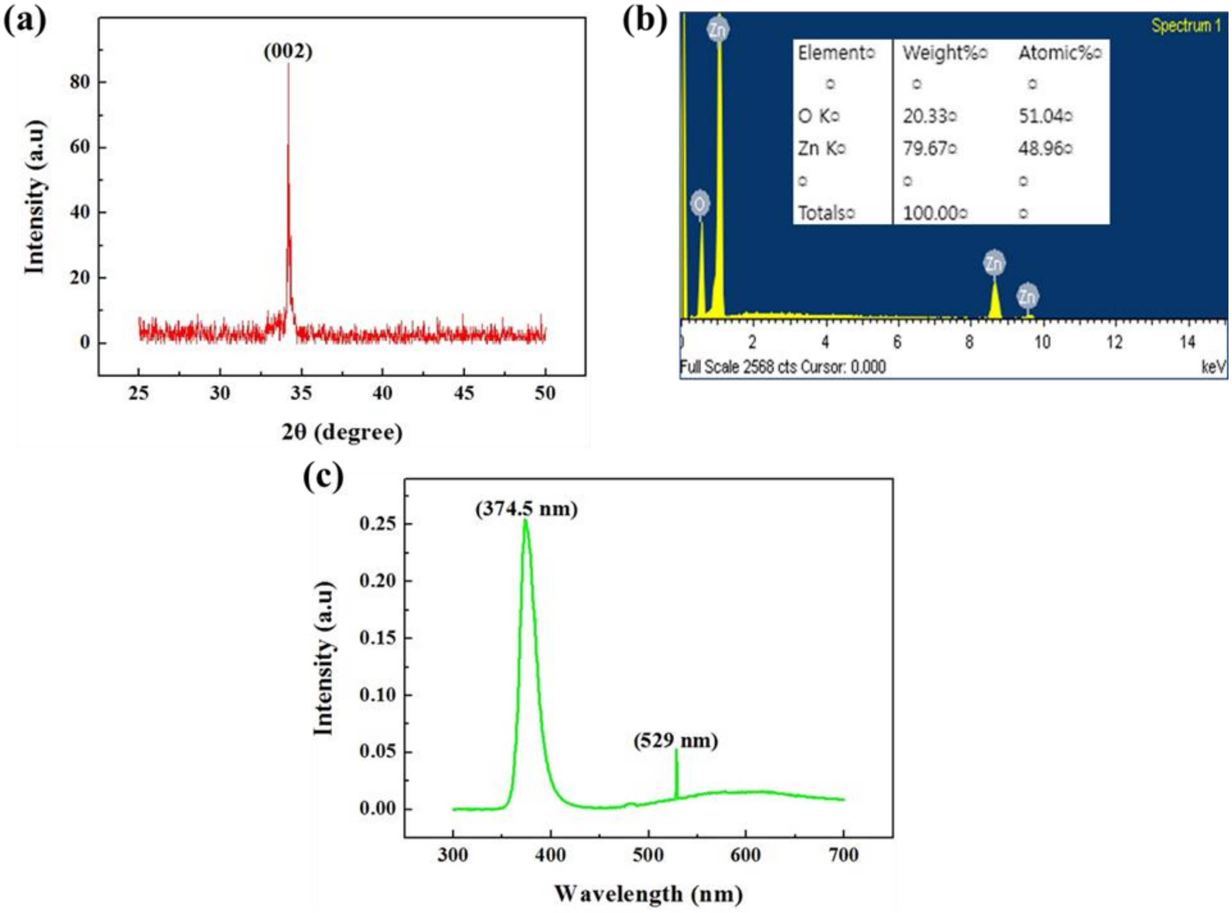

Figure S3. (a) XRD, (b) EDS, and (c) PL spectra of ZNRs grown with the microwave-assisted preheating method.
